# How Hosts Taxonomy, Trophy, and Endosymbionts Shape Microbiome Diversity in Beetles

**DOI:** 10.1007/s00248-019-01358-y

**Published:** 2019-03-27

**Authors:** Michał Kolasa, Radosław Ścibior, Miłosz A. Mazur, Daniel Kubisz, Katarzyna Dudek, Łukasz Kajtoch

**Affiliations:** 1 Institute of Systematics and Evolution of Animals, Polish Academy of Sciences, Krakow, Poland; 2 Department of Zoology and Animal Ecology, University of Life Sciences in Lublin, Lublin, Poland; 3 Institute of Biology, University of Opole, Opole, Poland; 4 Molecular and Behavioral Ecology Group, Jagiellonian University, Krakow, Poland

**Keywords:** Bacterial community, Host–microbe interactions, Coleoptera, Endosymbionts, Microbial ecology

## Abstract

Bacterial communities play a crucial role in the biology, ecology, and evolution of multicellular organisms. In this research, the microbiome of 24 selected beetle species representing five families (Carabidae, Staphylinidae, Curculionidae, Chrysomelidae, Scarabaeidae) and three trophic guilds (carnivorous, herbivorous, detrivorous) was examined using 16S rDNA sequencing on the Illumina platform. The aim of the study was to compare diversity within and among species on various levels of organization, including evaluation of the impact of endosymbiotic bacteria. Collected data showed that beetles possess various bacterial communities and that microbiota of individuals of particular species hosts are intermixed. The most diverse microbiota were found in Carabidae and Scarabaeidae; the least diverse, in Staphylinidae. On higher organization levels, the diversity of bacteria was more dissimilar between families, while the most distinct with respect to their microbiomes were trophic guilds. Moreover, eight taxa of endosymbiotic bacteria were detected including common genera such as *Wolbachia*, *Rickettsia*, and *Spiroplasma*, as well as the rarely detected *Cardinium*, *Arsenophonus, Buchnera*, *Sulcia*, *Regiella*, and *Serratia*. There were no correlations among the abundance of the most common *Wolbachia* and *Rickettsia*; a finding that does not support the hypothesis that these bacteria occur interchangeably. The abundance of endosymbionts only weakly and negatively correlates with diversity of the whole microbiome in beetles. Overall, microbiome diversity was found to be more dependent on host phylogeny than on the abundance of endosymbionts. This is the first study in which bacteria diversity is compared between numerous species of beetles in a standardized manner.

**Electronic supplementary material:**

The online version of this article (10.1007/s00248-019-01358-y) contains supplementary material, which is available to authorized users.

## Introduction

Multicellular organisms share an inextricable and mutualistic relationship with a large number of resident microorganisms, collectively known as the microbiota (microbiome) [1]. Nowadays, microbiome is considered one of the most important factors that shapes the life history of its hosts. Naturally, the best studied animal in the context of the relationship between microbiota and host is the human (e.g., [2]) and model organisms (e.g., [3–6]). In contrast, microbiome diversity and relations with hosts have rarely been investigated in the context of wild animals, but this is quickly changing thanks to the development of new sequencing technologies and their decreasing costs. Thus far, the majority of microbiome studies in relation to hosts have been conducted with vertebrates (e.g., [7–11]). It is assumed that the complexity and diversity of the symbioses of bacteria and invertebrates (particularly insects) are lower than those associated with vertebrates [12]. Simultaneously, the influence of bacteria on insect hosts should be similar. It has been proven that some organisms may have a significant influence on the composition of hosts microbiota: parasites [13], symbionts [14], or endosymbionts [15]. While interaction between host and parasite or symbionts are well-studied and documented [16, 17], scientists have only begun to clarify the relationship with endosymbionts. One of the most thoroughly examined groups of microbes are endosymbiotic bacteria belonging to so-called male-killing bacteria [18]. Endosymbiotic bacteria are a specific group of organisms that are known first of all to influence host reproduction. Several intracellular bacteria are known to have such an impact on their hosts. The best known are two genera of α-proteobacteria: *Wolbachia* and *Rickettsia*. Others, such as *Spiroplasma* (Mollicutes) and *Cardinium* (Bacteroidetes), are much less studied [19–22]. *Wolbachia* have been reported as being found in arthropods and filarial nematodes around the world [23, 24]. *Rickettsia* also seem to be a common bacterial symbiont of arthropods [25]; they can manipulate host reproduction in arthropods through various mechanisms (see [18, 26] for the review). Moreover, *Wolbachia* may be an obligatory bacteriocyte-associated nutritional mutualist [27], which highlights a previously unknown aspect of the parasitism-mutualism evolutionary continuum.

Although we have begun to better understand the importance and function of microbiota, the relationship between the diversity of microbiome and traits (both phylogenetic and ecologic) of their host remain understudied topics. Due to richness of species, well-studied taxonomic relationships, and a relatively low complexity level of microbiota, insects seemed to be the perfect choice for such studies. Actually, the relationship between host taxonomy, trophism, and microbiota has already been the subject of meta-analysis [28]. Nonetheless, the latter work summarized previous studies, which caused numerous limitations. The study did not allow for a full elaboration of the topic. First of all, because Colman’s work was based on meta-analysis, examined data could not be standardized. Secondly, that unintended sampling caused an overrepresentation of insect species from some orders (e.g., Hymenoptera); also, some trophic guilds were highly underrepresented (e.g., carnivorous and herbivorous). For example, coleopterans were included as representatives of Cerambycidae, Buprestidae and Scolytinae (xylophagous), Carabidae (carnivorous), and Tenebrionidae (omnivorous), but most of these groups were represented by single species. Third, the number of examined specimens per species was various but generally too low for proper estimation of microbiome diversity. Finally, this study was done before 2012, the period in which capabilities of molecular tools were limited and therefore, all bacteria sequences were generated using traditional Sanger sequencing in conjunction with molecular cloning. This could cause several biases, mainly leading to underrepresentation of microbiome diversities. An interesting study by Yun et al. [29] examines microbiome of more than 300 insect species with use of next-generation sequencing (NGS). This study focused on a wide coverage of hosts with very low sampling per species (mostly single specimens were used), which prevented any intraspecific analyses and allowed for comparison of microbiomes only on high taxonomic levels. Studies by Colman et al. [28] and Yun et al. [29] develop interesting topics, but to fully address relations between microbiota and their insect hosts, more detailed and restricted research are needed. For the purposes of this study, beetles were selected: this group gives numerous opportunities to study various relations between microbiome and hosts of various phylogenetic relations and ecological affinity.

Beetles are the most species-rich and diversified order of insects in the world, including approximately nearly 400,000 known species [30]. They can be found in most terrestrial and freshwater habitats. Members of Coleoptera belong to all major trophic guilds known in animals. Despite such complexity and diversity, knowledge of microbiota in this group of insects is limited. So far, microbiome has only been examined in single species belonging to one of the trophic guilds: detritivores–coprophages [31, 32], scavengers [33], xylophages or cambiophages [34–38], herbivores [39–42], as well as carnivores [43–45]. Complex analyses concerning diversity of microbiota in relation to beetle phylogeny (taxonomy) and ecology (e.g., trophy) has yet to be conducted. The only well-studied bacteria of beetles are endosymbiotic “male-killing” bacteria, particularly *Wolbachia* [22], which additionally makes Coleoptera a good group to choose for microbiome studies.

The aim of this study was to use NGS to characterize the microbiome composition of 24 beetle species belonging to three trophic guilds (detritivorous, herbivorous, carnivorous) and five families (Carabidae, Staphylinidae, Chrysomelidae, Curculionidae, Scarabaeidae). This part aimed to verify whether microbiota composition is more similar within the species than between hosts (H1) and was assessed on two levels: that microbiome is similar in beetles belonging to the same taxonomic unit (family) (H2) or trophic guild (H3). Secondly, we established whether infection by endosymbionts (mainly *Wolbachia* and *Rickettsia*) could influence composition of microbiome to verify the hypothesis that the presence of endosymbionts correlates with microbiota diversity (that an increased number of endosymbionts decreases number of other bacteria) (H4). Additionally, we checked if an abundance of endosymbionts are negatively correlated with each other in order to verify the hypothesis that particular genera of these bacteria occur interchangeably (H5).

## Materials and Methods

### Species Selection and Sampling

For the purposes of this study, 24 species of beetles were selected. Beetle species were chosen based on the following criteria: (i) taxonomic position, (ii) trophic assignment, and (iii) status of endosymbiotic bacterial infection (based on previous studies). The first and second categories were partially related, as in beetles most taxonomic units (e.g., families or genera) are strictly associated with a particular diet and foraging mode (e.g., the majority of Carabidae are carnivorous). We decided to analyze three basic trophic guilds known in beetles, that is: (1) carnivorous, (2) herbivorous, and (3) detrivorous (specifically coprophagous) species. Among carnivorous, members of two groups were selected: Carabidae (ground beetles) and Staphylinidae (rove beetles). Among herbivorous species, two groups were also selected: Curculionidae (weevils) and Chrysomelidae (leaf beetles). Finally, all detrivorous species were recruited from Scarabaeidae (dung beetles). To omit biases, which could be potentially caused by different habitat preferences, foraging, or distribution of sampled species, selected beetles (within trophic guilds and taxonomic units) were selected from species of similar habitat and food preferences. For example, all carnivorous (Carabidae and Staphylinidae) were required from riparian beetles, all herbivorous (Curculionidae and Chrysomelidae) from grassland species, and all detrivorous (Scarabaeidae) from coprophagous species associated with wild and domestic mammals. Considering restrictions of next-generation sequencing, the total number of specimens planned to be analyzed (200) were divided between taxonomic groups (families) and trophic guilds. In species selection, the third criterion was also included: infection status by endosymbiotic bacteria, particularly *Wolbachia*. Infection status was based on previous research with use of genotyping by multilocus sequence typing [46] (details in: [47–50]). Half of the species from each group (each family and each trophic guild) was selected to be infected by *Wolbachia*; the second half had to be uninfected. Finally, 24 beetle species were selected. For 16 species (four ground beetles, four rove beetles, four weevils and four leaf beetles), 10 specimens per species were randomly selected from the collection of beetles from Europe preserved for molecular studies in the Department of Collections Institute of Systematics and Evolution of Animals, Polish Academy of Sciences in Krakow. The remaining eight species of dung beetles were sampled on five random specimens each (the lower number of specimens was due to fewer sampling of beetles belonged to this group (difficulties in sampling larger number of specimens in the field). Details related to sampling (taxonomic affinity, trophy assignment and *Wolbachia* infection status of selected beetle species with number of analyzed specimens) are presented in Table 1.

**Table 1:** Basic information about sampled beetles with their taxonomic affinity, assignment to trophic guilds, sampling sites and dates, number of examined specimens and data about *Wolbachia* infection status (based on [50]

Species	Symbol	Family	Trophic guild	Sampling sites	Collection date	No isolates	No analyzed samples	*Wolbachia* Sanger seq	Sequence read archive accession numbers
*Bembidion decorum*	Bdec	Carabidae	Carnivorous	Poland, Raba river	2017	10	9	–	SAMN10518078-SAMN10518087
*Bembidion modestum*	Bmod	Carabidae	Carnivorous	Poland, Raba river	2017	10	10	–	SAMN10518088-SAMN10518097
*Bembidion punctulatum*	Bpun	Carabidae	Carnivorous	Poland, Raba river	2017	10	10	+	SAMN10518058-SAMN10518067
*Bembidion varicolor*	Bvar	Carabidae	Carnivorous	Poland, Raba river	2017	10	10	+	SAMN10518068-SAMN10518077
*Paederus limnophilus*	Plim	Staphylinidae	Carnivorous	Poland, Raba river	2017	10	10	+	SAMN10518248-SAMN10518257
*Paederus riparius*	Prip	Staphylinidae	Carnivorous	Poland, Raba river	2017	10	10	+	SAMN10518238-SAMN10518247
*Paederidus rubrothoracicus*	Prub	Staphylinidae	Carnivorous	Poland, Raba river	2017	10	9	–	SAMN10518228-SAMN10518237
*Paederidus ruficollis*	Pruf	Staphylinidae	Carnivorous	Poland, Raba river	2017	10	10	+	SAMN10518218-SAMN10518227
*Argoptochus quadrisignatus*	Aqua	Curculionidae	Herbivorous	Poland, Nida Basin	2016	10	10	–	SAMN10518168-SAMN10518177
*Centricnemus leucogrammus*	Cleu	Curculionidae	Herbivorous	Poland, Nida Basin	2016	10	7	–	SAMN10518158-SAMN10518167
*Eusomus ovulum*	Eovu	Curculionidae	Herbivorous	Poland, Nida Basin	2016	10	10	+	SAMN10518138-SAMN10518147
*Polydrusus inustus*	Pinu	Curculionidae	Herbivorous	Poland, Nida Basin	2016	10	10	+	SAMN10518148-SAMN10518157
*Aphthona venustula*	Aven	Chrysomelidae	Herbivorous	Poland, Nida Basin	2016	10	10	+	SAMN10518118-SAMN10518127
*Cheilotoma musciformis*	Cmus	Chrysomelidae	Herbivorous	Poland, Nida Basin	2016	10	10	–	SAMN10518128-SAMN10518137
*Crioceris duodecimpunctata*	Cduo	Chrysomelidae	Herbivorous	Poland, Nida Basin	2016	10	10	–	SAMN10518108-SAMN10518117
*Crioceris quatuordecimpunctata*	Cqua	Chrysomelidae	Herbivorous	Poland, Nida Basin	2016	10	10	+	SAMN10518098-SAMN10518107
*Aphodius depressus*	Adep	Scarabaeidae	Detritivorous	Bulgaria, Balkan Mts.	2015	5	5	–	SAMN10518198-SAMN10518202
*Aphodius haemorrhoidalis*	Ahae	Scarabaeidae	Detritivorous	Bulgaria, Balkan Mts.	2015	5	5	+	SAMN10518188-SAMN10518192
*Aphodius pusillus*	Apus	Scarabaeidae	Detritivorous	Bulgaria, Balkan Mts.	2015	5	5	–	SAMN10518183-SAMN10518187
*Aphodius sphacelatus*	Asph	Scarabaeidae	Detritivorous	Bulgaria, Balkan Mts.	2015	5	5	–	SAMN10518208-SAMN10518212
*Onthophagus ruficapillus*	Oruf	Scarabaeidae	Detritivorous	Bulgaria, Balkan Mts.	2015	5	5	+	SAMN10518193-SAMN10518197
*Onthophagus similis*	Osim	Scarabaeidae	Detritivorous	Bulgaria, Balkan Mts.	2015	5	5	+	SAMN10518203-SAMN10518207
*Onthophagus taurus*	Otau	Scarabaeidae	Detritivorous	Bulgaria, Balkan Mts.	2015	5	5	+	SAMN10518178-SAMN10518182
*Onthophagus ovatus*	Oova	Scarabaeidae	Detritivorous	Bulgaria, Balkan Mts.	2015	5	5	–	SAMN10518213-SAMN10518217

### DNA Isolation and Sequencing

DNA was isolated from 200 specimens belonging to 24 species of coleopterans using The Wizard Genomic DNA Purification Kit (Promega). Prior to isolation, all beetle specimens were surface-bleached in distilled water. Isolation was conducted in blocks with two blank samples for each block to rule out contamination with bacterial DNA. DNA was used within a few hours after isolation and then stored at − 20 °C. All laboratory procedures were carried out in accordance with the Earth Microbial Project protocol (http://www.earthmicrobiome.org/protocols-and-standards/16s/). The V4 region of the 16S SSU rDNA was amplified with 515FB-806RB primer pair: forward: GTGYCAGCMGCCGCGGTAA; reverse: GGACTACNVGGGTWTCTAAT [51] with the addition of an index sequence unique for each specimen and Illumina adaptor. PCR was performed using the following steps: 94 °C for 3 min and 35 cycles of 94 °C for 45 s, 50 °C for 60 s, and 72 °C for 90 s with a final extension of 72 °C for 10 min. Blank samples were run on agarose gel along with samples with DNA isolates. Since blank samples did not show any products on the gel, they were excluded from further procedures. PCRs with DNA of all samples were repeated three times to avoid batch effect [52]. Subsequently, three samples per specimen were pulled together and run on a 2% agarose gel with a 100-bp ladder to check for amplification efficacy. Samples were pooled equimolarly based on the intensity of the bands. The next pool of samples were run on gel through electrophoresis. Afterward, bands of desired length were cut out from the gel and cleaned with the Zymoclean Gel DNA Recovery Kit (Zymo). Concentration of the library was measured with Qubit and sequenced with Illumina MiSeq platform (600 cycles) using the MiSeq Reagent Kit v3.

### Pre-processing and Data Analysis

Demultiplexed paired-end fastq files generated by Illumina and a mapping file were used as input files. Sequences were pre-processed, quality filtered, and analyzed using QIIME2 version 2018.2 [53]. The DADA2 software package within QIIME2 was used for modeling and correcting Illumina sequenced fastq files including the removal of chimeras using the “consensus” method. Due to a drop of quality scores sequences were truncated 40 bases from forward and 70 bases from reverse reads (with Phred score 25 as a threshold). For taxa comparison, relative abundances based on all obtained reads were used. The QIIME2 q2-feature-classifier plugin were used. The Naïve Bayes classifier that was trained on the SILVA132 99% OTUs full-length sequence database and subsequent sequences from our dataset were assigned to OTUs from SILVA database with similarity on the 99% level. To view the taxonomic composition of the samples, the QIIME2 taxa bar plot command was used. Next, all unassigned sequences were excluded from further analysis. Alpha and beta-diversity analyses were performed with the q2-diversity plugin in QIIME2 at a sampling depth of 2000, which was based on rarefaction curve. Alpha diversity was calculated by Shannon’s diversity index (hereafter Shannon), observed OTUs (hereafter OTU), Pielou’s measure of species evenness (hereafter Pielou), and Faith’s phylogenetic diversity (hereafter Faith). For description of the particular bacteria present in higher number of reads in analyzed beetles, bacterial OTUs were pulled to the level of genus (or any higher taxonomic rank if lower classification was not possible), e.g., all OTU classified as *Wolbachia* were considered jointly, etc. Beta diversity was estimated with use of unweighted UniFrac (hereafter uwUniFrac) and weighted UniFrac (hereafter wUniFrac), Bray–Curtis distance (hereafter Bray–Curtis), and Jaccard distance (hereafter Jaccard). Basic statistics showing means, ranges and standard deviations of these indices were calculated for all examined species separately, as well as for families and trophic guilds. Differences in alpha diversity indices between groups of beetles on three levels—(i) among species from particular family, (ii) among families, and (iii) among trophic guilds—were statistically assessed with use of Kruskal-Wallis test (for which all the assumptions have been verified), which was next visualized in QIIME2. Beta diversity was compared between groups using principal coordinates analysis (PCoA) with pairwise comparisons (PERMANOVA) and visualized with the emperor plugin in QIIME2. The bacterial communities associated with three levels of grouping were ordinated, according to microbiome composition similarity, using the distance-based non-metric multi-dimensional scaling (NMDS) [54]. The differences among the bacterial communities associated with three levels of differentiation (species, family, trophy) were estimated by a nonparametric one-way analysis of similarity (ANOSIM) [55].

Moreover, endosymbionts abundance was correlated with modified alpha diversity indices. Abundance was expressed by number of reads found for *Wolbachia* and *Rickettsia*, as only these taxa were found to be numerous in obtained microbiome sequences. To avoid biases caused by self-explanation of microbiome diversity by the endosymbiont prevalence parameter (as endosymbiotic bacteria constituted a large part of bacteria in many samples), all reads belonging to *Wolbachia* and *Rickettsia* were removed from datasets before calculation of modified alpha diversity indices in this step of analyses. Additionally, correlations between number of reads found for all identified endosymbiotic bacteria were also calculated. Abovementioned correlation were also repeated on only presence–absence data of particular endosymbiotic taxa.

Due to the simplicity of analyzed dataset of beetle hosts (24 species from only five families and three trophic guilds), a high correlation between these three levels of sample assignment (species, family, and trophy) was observed (Rho = 0.98 between species and family, Rho = 0.95 between family and trophy, and Rho = 0.93 between species and trophy). This ruled out the possibility of any multivariate analyses on these three states of host assignment. However, the effects of host phylogenetic relations and abundance of endosymbionts on overall diversity of bacteria was estimated with use of generalized linear models (GLMs). Host phylogenetic relations were expressed by use of genetic distances (adopting Kimura-two parameters as the substitution model; [56] calculated from sequences of cytochrome oxidase gene, subunit I (coxI) obtained from beetles (data from [50]), hereafter Distance. Endosymbiotic bacteria prevalence was measured as a summarized number of 16S reads assigned to any bacteria from genera known as to be endosymbiotic for insects (see “Results” for details), hereafter Endosymbionts. Explained variable (microbiome diversity) was taken from QIIME2 calculations of alpha diversity (four separate GLMs were analyzed for Shannon, OTUs, Pielou, and Faith). To avoid biases caused by self-explanation of microbiome diversity by endosymbiont (this concern most abundant *Wolbachia* and *Rickettsia*) prevalence parameter (as endosymbiotic bacteria constituted a large part of bacteria in many samples), all reads belonged to endosymbiotic bacteria were removed from dataset before calculation of alpha diversity indices. Models were next ranged according to Akaike information criterion (AIC) and AIC weights (*w*) following recommendations of Burnham and Andreson [57]. Statistical analyses (except these from QIIME2) were executed in R package [58].

## Results

### General Characteristic of Microbiomes

After quality control, we obtained a total of 9,320,053 demultiplexed sequences from 200 specimens of 24 beetle species belonging to five families and three trophic guilds (Suppl. Table 1). The mean sequence frequency was 46,600.3 per specimen (median 31,316.0, min 0.0, max 368,452.0 per specimen). Five specimens (one *Paederidus rubrothoracicus*, one *Bembidion decorum*, and three *Centricnemus leucogrammus*) were excluded in this process.

After rarefaction on average, for most of specimens, 2000–4000 Illumina reads of 16S sequences were obtained; however, these values varied greatly (Table 2). The number of OTUs obtained from collected reads also varied greatly between particular beetles, but on average, 80–140 OTUs were observed per species (Table 2).

**Table 2:** Basic characteristics of collected data (sequences of bacteria) obtained from examined beetles. *N* reads = number of 16S rDNA reads; OTU = number of operational taxonomic units

Hosts	*N* reads				OTU			
	Mean	Min	Max	SD	Mean	Min	Max	SD
Species								
*Bembidion decorum*	41,535	800	117,373	37,915	195	10	491	148
*Bembidion modestum*	34,446	16,651	65,357	15,356	108	12	189	64
*Bembidion punctulatum*	33,125	12,089	140,466	38,979	164	67	285	71
*Bembidion varicolor*	22,052	11,885	48,390	12,678	165	38	398	122
*Paederus limnophilus*	91,999	63,990	127,029	21,732	46	11	173	60
*Paederus riparius*	30,512	11,733	47,180	11,340	29	7	71	24
*Paederidus rubrothoracicus*	31,033	13,231	54,406	14,539	27	8	58	16
*Paederidus ruficollis*	30,136	16,767	53,419	11,765	67	9	196	67
*Aphthona venustula*	33,882	2900	73,418	20,064	121	14	335	112
*Crioceris duodecimpunctata*	21,593	8003	34,031	9010	153	72	325	89
*Crioceris quatuordecimpunctata*	37,369	18,832	106,314	25,269	45	7	343	105
*Cheilotoma musciformis*	53,582	21,661	117,717	36,263	17	5	40	11
*Argoptochus quadrisignatus*	35,238	13,546	79,716	18,816	36	9	79	24
*Centricnemus leucogrammus*	5805	963	17,373	5693	73	29	124	31
*Eusomus ovulum*	53,938	17,797	105,669	24,506	108	5	348	122
*Polydrusus inustus*	26,859	6225	72,234	18,471	34	6	123	36
*Aphodius depressus*	226,814	135,860	368,452	86,183	207	150	270	50
*Aphodius haemorrhoidalis*	133,394	92,578	210,402	44,863	211	167	262	42
*Aphodius pusillus*	36,966	14,277	80,054	27,131	149	79	221	57
*Aphodius sphacelatus*	13,764	5041	22,026	6798	53	32	74	16
*Onthophagus ruficapillus*	95,836	85,436	118,802	14,673	173	77	219	60
*Onthophagus similis*	113,431	71,700	145,375	34,800	157	65	199	56
*Onthophagus taurus*	68,911	53,096	79,916	9779	201	156	237	35
*Onthophagus ovatus*	16,059	11,504	24,406	5195	107	81	139	26
Families								
Carabidae	32,789	800	140,466	28,702	158	10	491	108
Staphylinidae	46,302	11,733	127,029	30,963	43	7	196	49
Curculionidae	31,092	963	105,669	24,734	63	5	348	72
Chrysomelidae	36,606	2900	117,717	26,385	84	5	343	102
Scarabaeidae	88,147	5041	368,452	76,049	157	32	270	66
Trophic guilds								
Carnivorous	86,782	5041	368,452	75,599	154	31	270	68
Herbivorous	33,906	963	117,717	25,735	74	5	348	89
Detrivorous	39,460	800	140,466	30,417	101	7	491	102

**Fig. 1:**
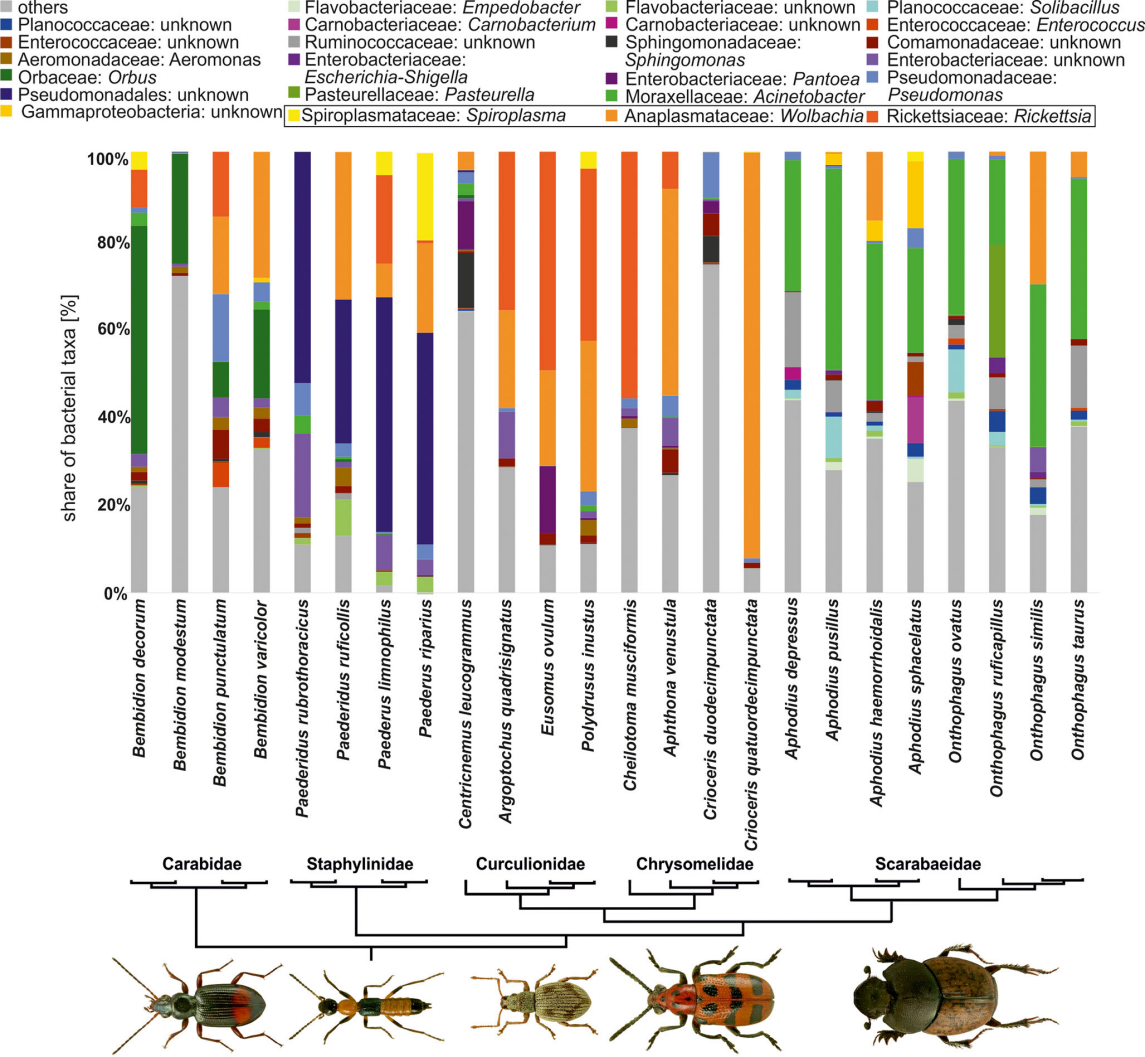
The plot of the relative share of bacteria in examined specimens of 24 species of beetles showed in relation to a simplified phylogenetic tree of examined beetles. Shown only bacterial operational taxonomic units, which relative frequencies higher than 5% and found in more than half of examined individuals in any of infected host. Photographs of exemplary infected beetle hosts were reprinted from ICONOGRAPHIA COLEOPTERORUM POLONIAE under a CC BY license, with permission (© Copyright. by Prof. Lech Borowiec, Wrocław 2007–2018, Department of Biodiversity and Evolutionary Taxonomy, University of Wroclaw, Poland)

Due to the high number of bacteria taxons within samples (Fig. 1, Suppl. Fig. 1), the descriptive part of results was focused on only bacteria, which OTUs were found in abundance (for at least one half of individuals from particular host species) higher than 5% (Suppl. Table 2). Occurrence of endosymbiotic bacteria (particularly *Wolbachia*, *Rickettsia*, and *Spiroplasma*) is described in following chapter; the descriptions below concern only other bacterial taxa.

Regarding ground beetles, more than 5% of the reads were found for one OTU of *Enterococcus* (Bacilli) in *Bembidion punctulatum*, one OTU from family Comamonadaceae (Betaproteobacteria) in *B. punctulatum*, two OTUs from Enterobacteriaceae and two OTUs from *Pseudomonas* (both Gammaproteobacteria) in *B. punctulatum*, one *Orbus* OTU (Gammaproteobacteria) in all *Bembidion* species and unidentified Proteobacteria OTU in *B. modestum* (Suppl. Table 2).

Among rove beetles, one OTU of Flavobacteriaceae (Flavobacteriia) was abundant in *Paederus ruficollis*, one Leptotrichiaceae OTU (Fusobacteriia) was found in *P. rubrothoracicus*, two OTUs of Enterobacteriaceae (Gammaproteobacteria) were present in *P. rubrothoracicus*, one OTU from order Pseudomonadales (both Gammaproteobacteria) infected all rove beetle species (Suppl. Table 2).

In weevils, one *Sphingomonas* OTU (Alphaproteobacteria) was found in *C. leucogrammus*, *Pantoea* OTU (Gammaproteobacteria) was numerous in *C. leucogrammus* and *Eusomus ovulum*, and one OTU from family Enterobacteriaceae (Gammaproteobacteria) was found in *Argoptochus quadrisignatus* (Suppl. Table 2).

Regarding leaf beetles, one OTU of *Sphingomonas* (Alphaproteobacteria) was found in *Crioceris duodecimpunctata*, Comamonadaceae OTU (Betaproteobacteria) was present in *Aphthona venustula* and *C. duodecimpunctata*, and Enterobacteriaceae OTU (Gammaproteobacteria) was found in *A. venustula* (Suppl. Table 2).

Among dung beetles, in all eight species, three *Acinetobacter* OTUs (Gammaproteobacteria) dominated. Moreover, the following bacteria reached 5% in some hosts: *Empedobacter* OTU (Flavobacteriia) in *Aphodius sphacelatus*, *Solibacillus* OTU (Bacilli) in *A. pusillus* and *Onthophagus ovatus*, *Carnobacterium* OTU (Bacilli) in *A. sphacelatus*, Enterococcaceae OTU (Bacilli) in *A. sphacelatus*, Ruminococcaceae OTU (Clostridia) in *A. depressus* and *O. taurus*, Neisseriaceae OTU (Betaproteobacteria) in *A. sphacelatus*, Enterobacteriaceae OTU (Gammaproteobacteria) in *O. similis*, *Pasteurella* OTU (Gammaproteobacteria) in *O. ruficapillus* and unidentified Gammaproteobacteria OTU in *A. sphacelatus* (Suppl. Table 2).

### Alpha Diversity

**Fig. 2:**
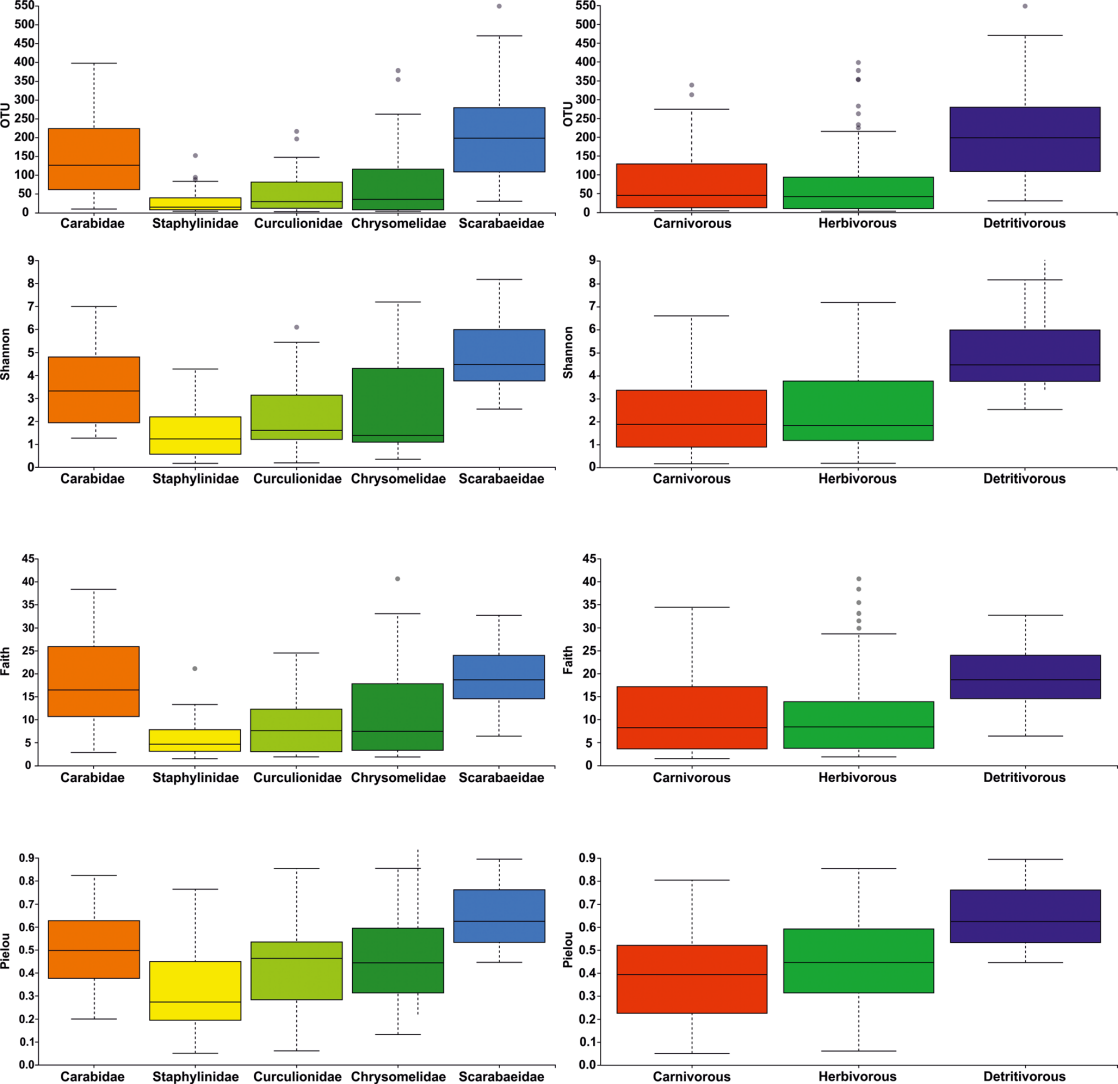
Box plots of microbiome alpha diversity metrics (observed operational taxonomic units = OTU, Shannon’s diversity index = Shannon, Faith’s Phylogenetic Diversity = Faith, and Pielou’s measure of species evenness = Pielou) presented for beetles from selected families (left panel) and trophic guilds (right panel)

Microbiome alpha diversity differed significantly among samples in all three levels of grouping (species, family, and trophic guild), and it was observed for all four metrics (OTU, Shannon, Faith, and Pielou) (Table 3, Suppl. Table 3a, Fig. 2, Suppl. Fig. 2).

**Table 3:** Results of statistical comparison of microbiome alpha diversity metrics (Kruskal–Wallis test) and beta diversity metrics (PERMANOVA) calculated on three levels: beetle species, families, and trophic guilds

Alpha diversity								
Kruskal–Wallis test	OTUs		Shannon		Faith		Pielou	
	*H*	*p* value	*H*	*p* value	*H*	*p* value	*H*	*p* value
Species	123.03	< 0.001	115.73	< 0.001	118.85	< 0.001	93.56	< 0.001
Family	79.67	< 0.001	74.10	< 0.001	71.68	< 0.001	54.53	< 0.001
Trophism	45.17	< 0.001	46.41	< 0.001	32.99	< 0.001	38.45	< 0.001
Beta diversity								
PERMANOVA	Bray–Curtis		Jaccard		Weighted UniFrac		Unweighted UniFrac	
	pseudo-*F*	*p* value	Pseudo-*F*	*p* value	Pseudo-*F*	*p* value	Pseudo-*F*	*p* value
Species	8.50	0.001	3.64	0.001	9.82	0.001	5.92	0.001
Family	14.15	0.001	6.89	0.001	16.77	0.001	15.45	0.001
Trophism	2,714,453.00	0.001	7.49	0.001	20.56	0.001	20.25	0.001

Among ground beetles, the highest microbiome diversity was found in *B. decorum* and *B. punctulatum* and lower in *B. modestum* and *Bembidion varicolor*. *P. ruficollis* had the highest microbiome diversity among all rove beetles with other three taxa of similarly lower metrics of diversity. In weevils, *C. leucogrammus* possessed most diverse bacteria, followed by *E. ovulum*, *Polydrusus inustus*, and *A. quadrisignatus*. Two sister species of *Crioceris* leaf beetles had totally different diversity of microbiome with *C. duodecimpunctata* of much higher diversity of bacteria. Also, *A. venustula* had much more diverse microbiome than *Cheilotoma musciformis*. Among dung beetles, most species were characterized by high diversity of bacteria, and only *A*. *sphacelatus* had very low microbiome diversity (Table 2, Suppl. Table 3a, Suppl. Fig. 2).

On the level of the beetle family, there were substantial differences in diversity of bacteria. Overall, the highest diversity of microbiome was observed for Scarabaeidae and next for Carabidae. Lower diversity was found for Chrysomelidae and Curculionidae and the lowest for Staphylinidae (Table 2, Suppl. Table 3a, Fig. 2).

Obviously, a similar pattern was observed for beetles grouped into trophic guilds, with highest diversity of bacteria found for detritivores and lowest diversities observed in herbivores and carnivores (the latter was caused by joint measuring of diversity of bacteria rich ground beetles and bacteria of poor rove beetles) (Table 2, Suppl. Table 3a, Fig. 2).

### Beta Diversity

**Fig. 3:**
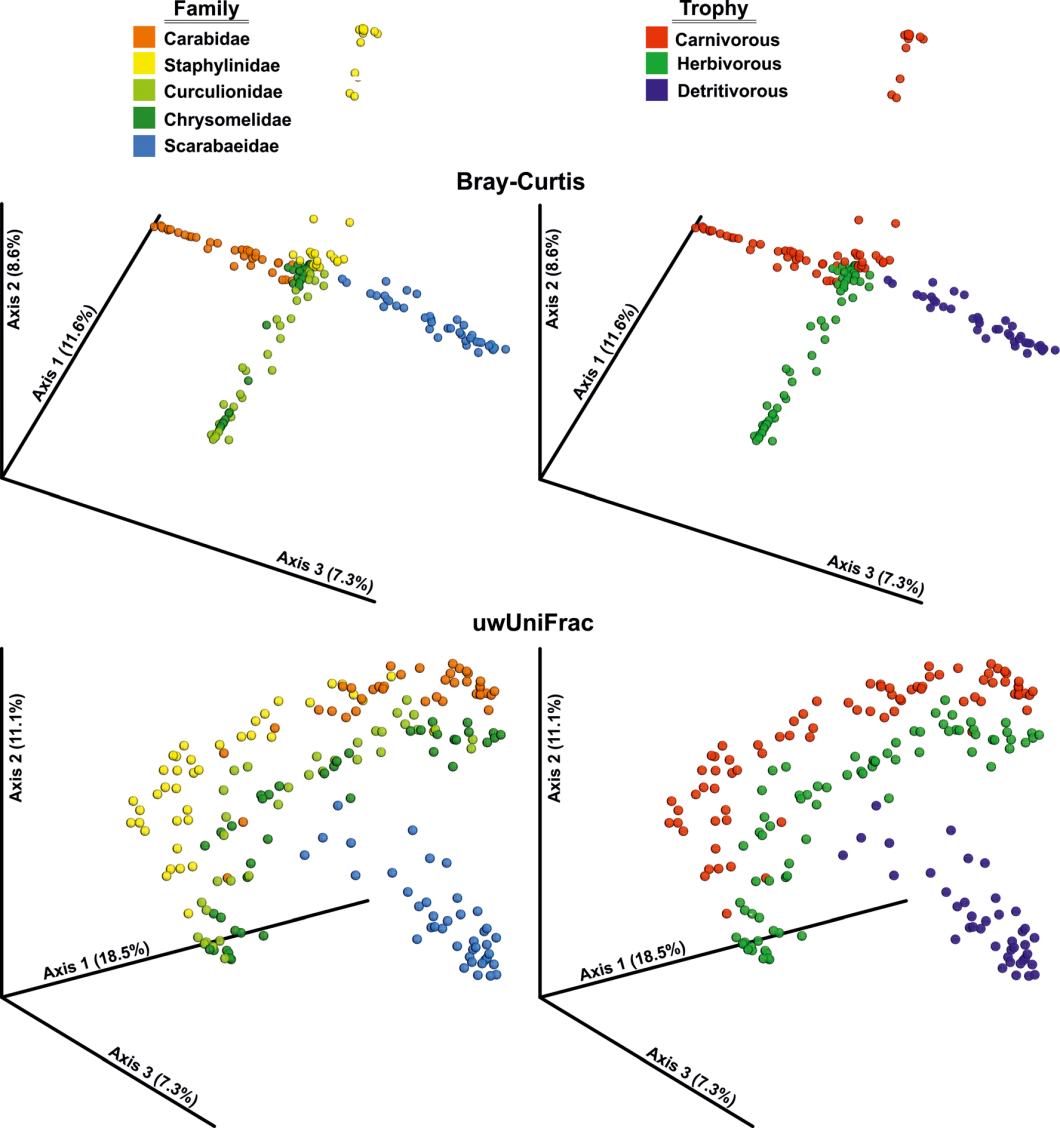
Principal coordinate analyses of microbiome beta diversity metrics (Bray–Curtis distance = Bray-Curtis and unweighted UniFrac = uwUniFrac) presented for beetles on the level of families and trophic guilds

All four metrics of beta diversity (Bray–Curtis, Jaccard, wUniFrac, and uwUniFrac) resulted in consistent results. However, distances between beetle hosts were found to be different depending on the level of species grouping: species, families or trophic guilds (Table 3, Suppl. Table 3b, Fig. 3, Suppl. Figs. 3, 4).

Regarding microbiome similarity between particular species of beetles, most hosts belonging to the same taxa did not cluster into distant groups from other members of either the same family or trophic guild. Microbiomes found in individuals of beetles generally were intermixed and only rarely did some beetle species separate from others (Suppl. Table 3b, Suppl. Fig. 3).

In Carabidae only *B. varicolor* formed a separate unit; however, single individuals of this species seemed to have microbiome more similar to *B. decorum* or *B. punctulatum*. Among Staphylinidae and Curculionidae, no species formed distinct units in respect to their microbiota. In Chrysomelidae, 8 out of 10 specimens of *Crioceris quatuordecimpunctata* were found to be weakly different. Also, in Scarabaeidae, four out of five specimens of *A.sphacelatus* were separated from all other specimens from this family (Suppl. Table 3b, Suppl. Fig. 3).

If considering trophic guilds, no beetle species formed clustered separately in carnivorous, herbivorous or detrivorous (Suppl. Table 3b, Suppl. Fig. 3).

Quite opposite patterns were found when analyzing beetles in groups. On the level of families (Table 3, Suppl. Table 3b, Fig. 3, Suppl. Fig. 4), the most distinct microbiome was found to have dung beetles. Also, ground beetles and rove beetles formed partially separate groups with some overlap between species from these two groups. The only exceptions seemed to be Curculionidae and Chrysomelidae, members of which intermixed in respect to distinctiveness of their microbiota.

**Fig. 4:**
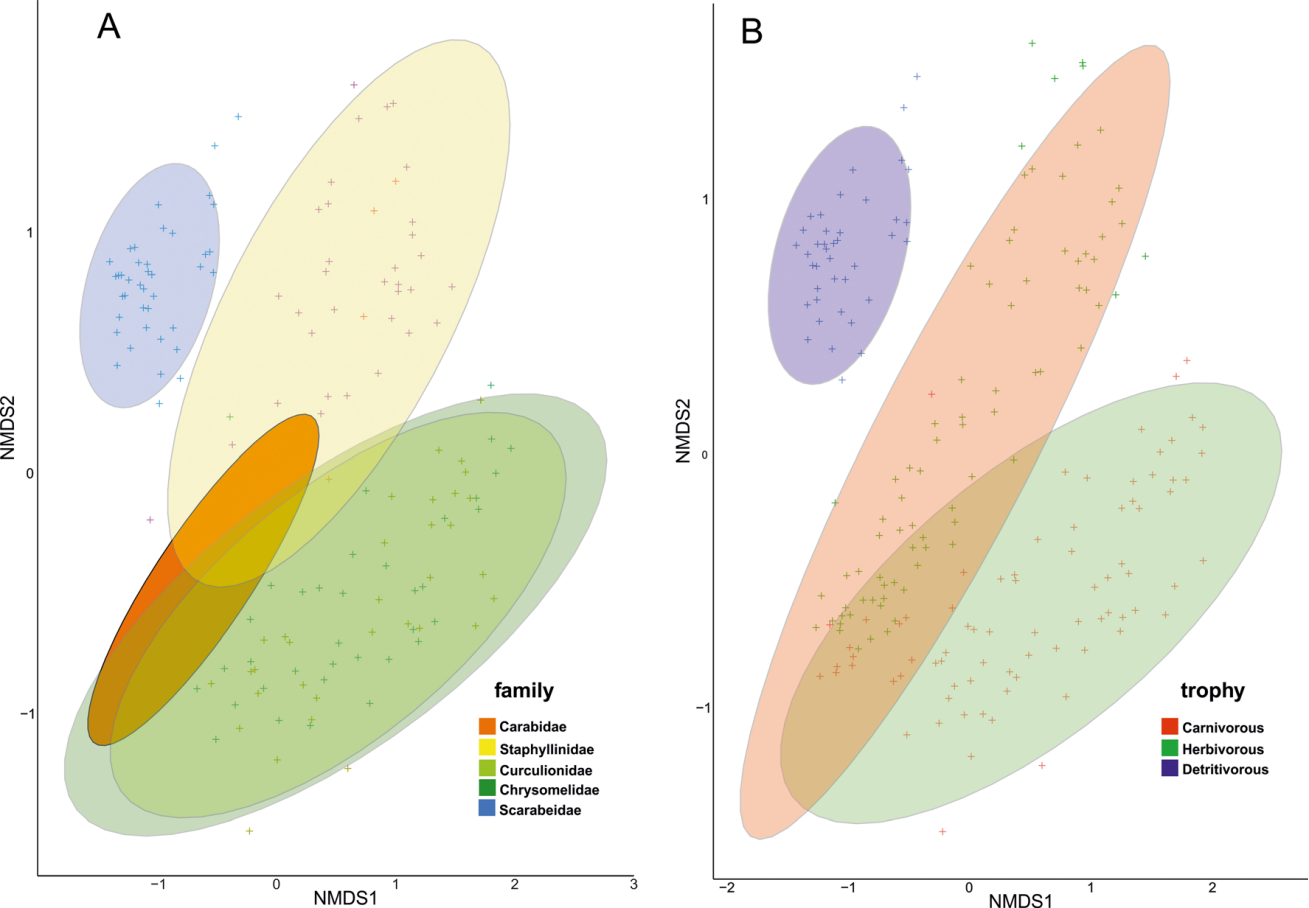
Non-metric multidimensional scaling plot of Bray-Curtis dissimilarities between microbiomes found in beetle individuals belonged to particular families (**a**) and trophic guilds (**b**). Ellipses = 95% confidence intervals.

The abovementioned patterns resulted in much more visible distinctiveness of microbiomes found in hosts belonging to particular trophic guilds (Table 3, Suppl. Table 3b, Fig. 3. Suppl. Fig. 4). All three guilds (carnivorous, herbivorous and detrivorous) formed separate groups, with detrivorous most distant to all others. ANOSIM revealed significant differences of microbiome similarities on all three levels of grouping (ANOSIM *R* = 0.837, *P* = 0.001 for species; *R* = 0.693, *P* = 0.001 for families and *R* = 0.590, *P* = 0.001 for trophic guilds). This was also supported by NMDS plots, which show that microbiome of beetles from particular trophic guilds and families are generally different with some exceptions (e.g., weevils and leaf beetles; Fig. 4). Less obvious pattern was observed for species level, as numerous beetles overlapped in NMDS plot (Supp. Fig. 5).

### Endosymbionts

In total, nine taxa of endosymbiotic bacteria (including five from the male-killing group) were observed in examined beetles (Table 4). The most abundant were found to be Rickettsiales with the highest share of *Wolbachia* and *Rickettsia*. *Wolbachia* was the dominant bacteria (in respect to number of obtained reads among the whole microbiome) in *B. punctulatum*, *B. varicolor*, *P. limnophilus*, *Paederus riparius*, *P. ruficollis*, *A. venustula*, *C. quatuordecimpunctata*, *A. quadrisignatus*, *E. ovulum*, *P. inustus*, *A. haemorrhoidalis*, *O. similis*, and *O. taurus*, in 13 out of 24 examined species. Three distinct OTUs were identified for *Wolbachia*. Their occurrence was generally host species-specific, but there are also cases in which two OTUs were present in the same species and even individual. In all such cases, one OTU over-dominating other (see Suppl. Table 1 for details).

**Table 4:** Occurrence of endosymbiotic bacteria (expressed as the relative share with respect to the whole microbiome = reads (%) and share of infected individuals = *N* (%)) found in examined species of beetles. SD = standard deviation. Symbols of beetle species follows Table 1

Host	Variable	*Buchnera*	*Regiella*	*Serratia*	*Sulcia*	*Arsenophonus*	*Cardinium*	*Spiroplasma*	*Wolbachia*	*Rickettsia*
*Bembidion decorum*	Reads ± SD (%)							0.2241 ± 0.0409	0.0007 ± 0.0001	0.8817 ± 0.2629
	*N* (%)							70	20	50
*Bembidion modestum*	Reads ± SD (%)							0.0014 ± 0.0002	0.0036 ± 0.0004	0.0010 ± 0.0002
	*N* (%)							50	50	30
*Bembidion punctulatum*	Reads ± SD (%)							0.0231 ± 0.0021	1.8155 ± 0.2622	1.7238 ± 0.2546
	*N* (%)							60	100	60
*Bembidion varicolor*	Reads ± SD (%)						0.0012 ± 0.0006	0.0594 ± 0.0178	3.4413 ± 0.4115	0.0009 ± 0.0002
	*N* (%)						20	10	60	20
*Paederus limnophilus*	Reads ± SD (%)					0.0062 ± 0.0019		3.2044 ± 0.3414	0.8484 ± 0.2543	2.0600 ± 0.3057
	*N* (%)					10		60	50	100
*Paederus riparius*	Reads ± SD (%)							4.9069 ± 0.3795	1.9545 ± 0.2585	0.0645 ± 0.0167
	*N* (%)							90	80	50
*Paederidus rubrothoracicus*	Reads ± SD (%)							0.0471 ± 0.0087	0.0008 ± 0.0002	
	*N* (%)							44	22	
*Paederidus ruficollis*	Reads ± SD (%)			0.0247 ± 0.0074				0.0544 ± 0.0145	3.8582 ± 0.3069	0.0004 ± 0.0001
	*N* (%)			10				20	100	10
*Aphthona venustula*	Reads ± SD (%)	0.0053 ± 0.0009							4.7091 ± 0.3068	0.9181 ± 0.2750
	*N* (%)	10							100	30
*Cheilotoma musciformis*	Reads ± SD (%)				0.0003 ± 0.0001			0.2512 ± 0.0730	13.4766 ± 0.1853	0.0106 ± 0.0021
	*N* (%)				10			30	100	20
*Crioceris quatuordecimpunctata*	Reads ± SD (%)								0.0010 ± 0.0002	7.7705 ± 0.2205
	*N* (%)								30	100
*Crioceris duodecimpunctata*	Reads ± SD (%)	0.2245 ± 0.0260	0.0617 ± 0.0161					0.0555 ± 0.0137	0.0040 ± 0.0007	0.0059 ± 0.0015
	*N* (%)	90	30					70	30	20
*Argoptochus quadrisignatus*	Reads ± SD (%)	0.0397 ± 0.0093						0.0039 ± 0.0012	3.1186 ± 0.3098	5.8905 ± 0.2815
	*N* (%)	30						10	70	100
*Centricnemus leucogrammus*	Reads ± SD (%)	0.0032 ± 0.0010		0.0410 ± 0.0056				0.0074 ± 0.0016	0.3944 ± 0.0958	0.0142 ± 0.0027
	*N* (%)	89		44				22	100	56
*Eusomus ovulum*	Reads ± SD (%)	0.0115 ± 0.0035	0.0122 ± 0.0036		0.0002 ± 0.0001			0.0029 ± 0.0009	2.2034 ± 0.2067	6.7219 ± 0.3372
	*N* (%)	10	10		10			60	100	90
*Polydrusus inustus*	Reads ± SD (%)							2.2160 ± 0.1828	3.3485 ± 0.1367	5.7383 ± 0.2453
	*N* (%)							100	100	100
*Aphodius depressus*	Reads ± SD (%)	0.0001 ± 0.0000						0.0015 ± 0.0006	0.0015 ± 0.0003	0.0006 ± 0.0002
	*N* (%)	20						20	60	40
*Aphodius haemorrhoidalis*	Reads ± SD (%]							0.0315 ± 0.0052	1.1689 ± 0.0998	0.0050 ± 0.0012
	*N* (%]							60	100	80
*Aphodius pusillus*	Reads ± SD (%)	0.0001 ± 0.0000						0.0434 ± 0.0167	0.0166 ± 0.0049	0.0109 ± 0.0026
	*N* (%]	20						40	80	100
*Aphodius sphacelatus*	Reads ± SD (%]							0.2496 ± 0.0970		
	*N* (%)							60		
*Onthophagus ruficapillus*	Reads ± SD (%)	0.0000 ± 0.0000		0.0210 ± 0.0084					0.0752 ± 0.0180	0.0023 ± 0.0006
	*N* (%)	20		20					100	60
*Onthophagus similis*	Reads ± SD (%)							0.0048 ± 0.0019	1.8367 ± 0.3061	0.0017 ± 0.0002
	*N* (%)							20	80	80
*Onthophagus taurus*	Reads ± SD (%)								0.7251 ± 0.1168	0.0002 ± 0.0000
	*N* (%)								80	40
*Onthophagus ovatus*	Reads ± SD (%)									0.0006 ± 0.0002
	*N* (%]									20

*Rickettsia* was most abundant in *B. decorum*, *B. punctulatum*, *P. limnophilus*, *C. musciformis*, *A. quadrisignatus*, *E. ovulum*, and *P. inustus*, in 7 out of 24 examined beetles. In four species (*B. punctulatum*, *P. limnophilus*, *E. ovulum*, and *P. inustus*) both these bacteria were found in high prevalence (Table 4). Two OTUs of *Rickettsia* were present in analyzed beetles, and one of them was found in much higher number of reads (see Suppl. Table 1 for details). Contrary to *Wolbachia*, cases with two OTUs present in the same species and individual were rare.

*Spiroplasma* found be much less numerous (in respect to Illumina reads), but it was found in quite large numbers in the following beetle hosts: *P. limnophilus*, *P. riparius*, *P. inustus*, and in lower number in, e.g., *B. decorum*, *C. quatuordecimpunctata*, *A*. *sphacelatus* (Table 4). There were four OTUs of *Spiroplasma* in the whole dataset, but one of these units was over-dominating and was found in the majority of beetle hosts with the exception of predatory species (see Suppl. Table 1 for details). Similarly, as in *Rickettsia*, there were almost no cases with multiple units present in the single species and individual.

Other six taxa of endosymbiotic bacteria, only small number of reads in single hosts were found, such as: *Cardinium* (in *B. varicolor*); *Arsenophonus* (in *P. limnophilus*); *Buchnera* (in *A. venustula*, *C. duodecimpunctata*, *A. quadrisignatus*, *C. leucogrammus*, *E. ovulum*, *A. depressus*, *A. pusillus*, *O. ruficapillus*); *Regiella* (*C. duodecimpunctata*, *E. ovulum*); *Serratia* (*P. ruficollis*, *C. leucogrammus*, *O. ruficapillus*); and *Sulcia* (*C. quatuordecimpunctata*, *E. ovulum*). Only for *Buchnera* were four OTUs identified. All other endosymbiotic bacteria were represented by a single OTU found in all infected beetles (see Suppl. Table 1 for details).

**Fig. 5:**
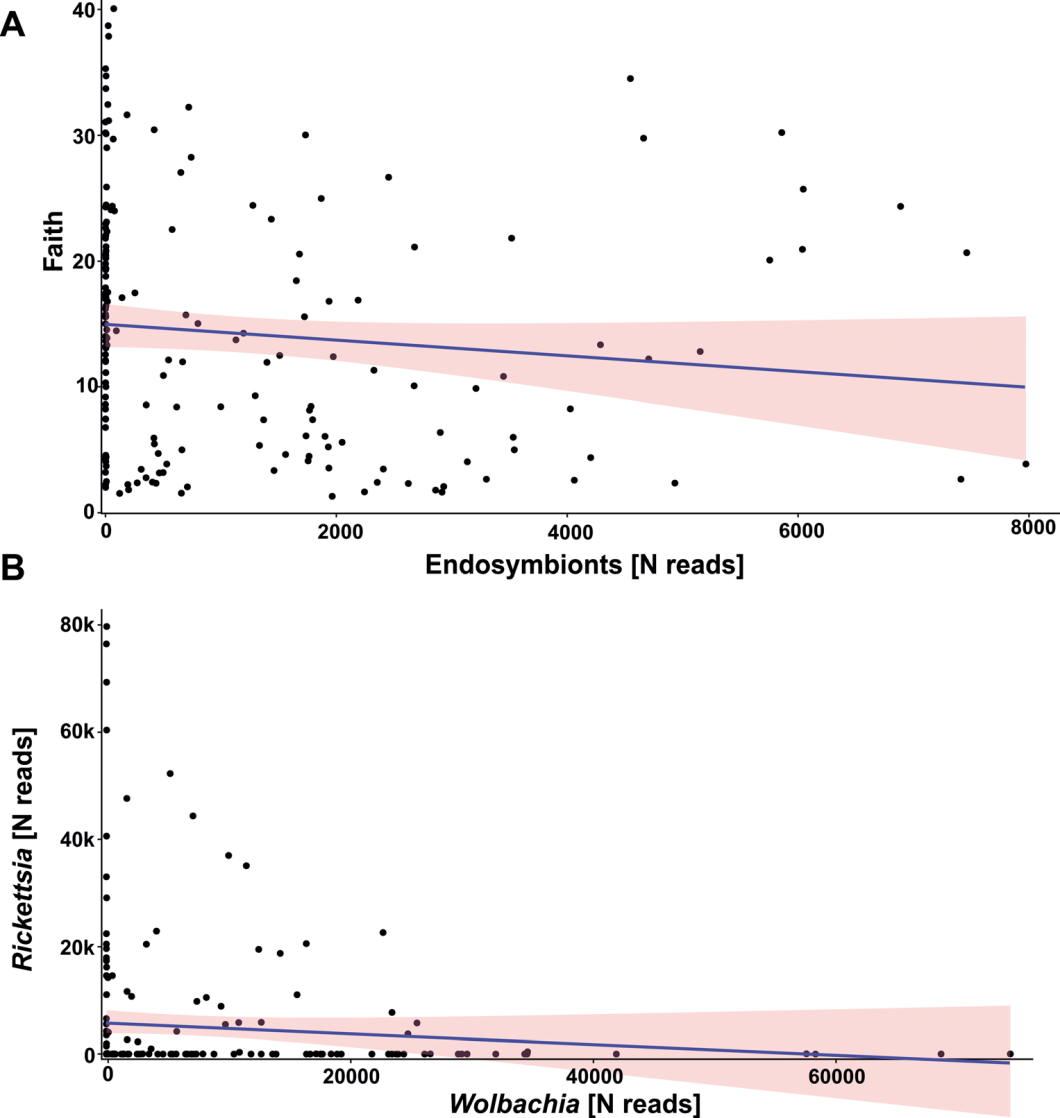
Correlation between **a** microbiome diversity (presented on example of Faith’s Phylogenetic Diversity) and endosymbiotic bacteria prevalence (numbers of Illumina reads of 16S rDNA sequences) in examined beetles and **b***Wolbachia* and *Rickettsia* prevalence

There were no significant correlations among number of reads of these endosymbiotic bacteria, with the exception of *Buchnera* and *Regiella* (Rho = 0.45 if consider number of reads and Rho = 0.50 for presence/absence data) as well as *Buchnera* and *Wolbachia* (Rho = − 0.17 only if considering number of reads). For the two most abundant endosymbiotic bacteria, i.e., *Wolbachia* and *Rickettsia*, no correlation was found (Rho = 0.04, *p* > 0.05 if consider number of reads and Rho = − 0.02, *p* > 0.05 for presence/absence data; Fig. 5a).

Regarding the endosymbiotic bacteria, their total number of reads significantly but very weakly and negatively correlated with alpha diversity of other bacteria in beetle hosts (Rho = − 0.21 for OTU, Rho = − 0.18 for Shannon, Rho = − 0.20 for Faith, and Rho = − 0.17 for Pielou Fig. 5b). If consider presence/absence data, significant but weak correlations were observed only between alpha diversity metrics and *Rickettsia* and *Buchnera* (e.g., Rho = − 0.29 and 0.21 for Faith, respectively). All other endosymbiotic bacteria were not correlated with any of alpha diversity metrics.

### Hosts Distances vs Endosymbionts

For three out of four alpha diversity metrics (Shannon, Faith, and Pielou), the model which was found to have the lowest AIC and highest AIC weight was that which included both explanatory variables, that is: Distance and Endosymbionts (Table 5). Only in the case of OTU, “the best” model included only Distance. However, in other GLMs (with the exception of Faith), the second model had AIC weight lower from the best model of less than 2, what according to Arnold [59] did not allow for recognizing which of these models better explained microbiome diversity. In all GLMs, the model with the lowest AIC weight included only Endosymbionts (Table 5), what indicated that abundance of endosymbionts was a much worse explanatory of microbiome diversity than genetic distances of infected hosts.

**Table 5:** Generalized linear models verifying which explanatory variable—cytochrome oxidase subunit I distances among infected beetles [Distance] or abundance of endosymbiotic bacteria [Endosymbionts]—better explain the alpha diversity metrics of microbiome found in examined beetles

Model	*k*	AIC	∆	*w*
OTU				
Distance	2	2229.0	0.00	0.56
Distance + endosymbionts	1	2229.7	0.62	0.41
Endosymbionts	1	2236.1	7.09	0.02
Intercept	1	2236.8	7.75	0.01
Shannon				
Distance + Endosymbionts	2	778.2	0.00	0.70
Distance	1	779.9	1.74	0.30
Endosymbionts	1	793.8	15.60	0.00
Intercept	1	797.0	18.75	0.00
Faith				
Distance + Endosymbionts	2	1584.8	0.00	0.98
Distance	1	1592.3	7.54	0.02
Endosymbionts	1	1628.2	43.39	0.00
Intercept	1	1642.2	57.39	0.00
Pielou				
Distance + Endosymbionts	2	6.1	0.00	0.72
Distance	1	8.0	1.84	0.28
Endosymbionts	1	23.0	16.84	0.00
Intercept	1	26.6	20.50	0.00

## Discussion

The most important discovery of the presented study is that microbiota communities vary greatly in insect hosts (here using beetles) and that bacteria diversity is shaped by both host phylogenetic relations (in simplicity taxonomy) and trophic affinity. The novelty of this study is that it simultaneously analyses microbiome of numerous taxa on several specimens each, whereas almost all former research [31, 33, 35] was focused on single representatives belonging to a particular taxonomic unit and trophic guild. According to a brief literature search, there are only several studies on microbiome of selected beetles (all listed in the “Introduction”). These previous studies did not allow for comparative analyses. In contrast, numerous specimens are examined in this study at the same time and conditions. They belonged to 24 selected species of beetles from five families and three trophic guilds. It also exceeds the pioneer works of Colman et al. [28] and Yun et al. [29], which were the only studies that examined microbiome diversity in respect to hosts taxonomy (for various insects) and diet preferences. However, the studies of Colman and Yun, which inspired our work, had some limitations listed above (see “Introduction”), which are overcome in the presented article.

Our results indicate that bacterial communities are highly diverse on every level of grouping. Regarding alpha diversity of bacteria found in beetle hosts, a high variability of microbiota in coleopterans was observed on all examined levels, starting from variable number of OTUs found in specimens of particular species as well as various values of diversity metrics on the level of families and trophic guilds. On the species level, it was interesting to detect different values of alpha diversity metrics even between closely related species (from the same genera), which share habitats and diet. Such a situation was observed for four *Bembidion* ground beetles (e.g., *B. decorum* has on average 2.5 times more diverse microbiome than *B. modestum*), *Aphodius* dung beetles (e.g., *A. depressus* has on average eight times more diverse microbiome than *A. sphacelatus*), and *Onthophagus* dung beetles (e.g., *O. taurus* has on average four times more diverse microbiome than *O. ovatus*). The most pronounced example is two sister *Crioceris* leaf-beetles [60], with *C. quatuordecimpunctata* c. 14-fold lower bacteria diversity than *C. duodecimpunctata*. Kolasa et al. [48] showed that these two species differ also in terms of *Wolbachia* infection (*C. quatuordecimpunctata* totally infected, *C. duodecimpunctata* uninfected), despite feeding on the same host plant. As *Wolbachia* infection could cause decreases of bacterial diversity (see [3] for *Drosophila melanogaster*), the high prevalence of this endosymbiont in *C. quatuordecimpunctata* could explain its very low diversity of the entire microbiome. The only exception seemed to be *Paederus/Paederidus* rove beetles, as all four examined species share similar and low levels of microbiome diversity. These differences in bacteria diversity among particular species of beetles are not easily explained due to deficiency of other studies for comparison. Some biases caused by laboratory procedures could not be totally ruled out. However, all conditions (during DNA isolation, amplification, and sequencing) were the same for each specimen. Also, the number of examined individuals were the same within families. It could not be simply explained by different microbiota associated with species hosts, as particular individuals of beetles, even within species, often expressed by very different composition of bacteria. It is interesting that on principal coordinate analyses plots particular specimens are usually intermixed between species. Contrary to this, bar plots (showing relative abundance of bacteria across hosts) suggest that particular beetle species is dominated by one, two, or few bacterial taxa. One explanation for these results could be that it suggests that lack of distinctiveness of microbiota found in members of particular beetle species is caused by a high diversity of low-abundant bacteria. Second possibility is that these few dominating bacterial taxa could be responsible for similarities of beetle hosts if considering them on higher levels (taxonomic or trophic). The majority of beetle species were found to be infected by widespread, usually aerobic bacteria of commensal lifestyle (e.g., *Enterococcus* (Bacilli), Comamonadaceae (Betaproteobacteria), Enterobacteriaceae and *Pseudomonas* (both Gammaproteobacteria), Neisseriaceae (Betaproteobacteria) etc.). *Enterococcus* is a member of Firmicutes, which were found to be dominant bacteria in insect microbiomes [29]. These aerobic bacteria are most common in ground beetles and rove beetles and constitute a substantial part of microbiome in weevils and leaf beetles as well. All four of these groups of beetles are free-living and are associated with various types of fresh foods, either animals (small invertebrates in case of carnivores) or plants (green tissues in case of herbivores). It is interesting that in rove beetles, a high share of bacteria were anaerobic Flavobacteriaceae, Fusobacteria, and Clostridia, which is consistent with the lifestyle of *Paederus/Paederidus*. These species occupy muddy riverbanks and live within decomposing remains of plants, where aerobic conditions are present. In some species of weevils, aerobic bacteria were present, which could be present on the surface of leaves of their host plants. The presence of these bacteria could be explained in light of findings of synergies in insect-microbe relationships at the interface of plant–insect defenses [39]. Dominant bacteria in many beetles were endosymbiotic taxa, which is concordant with microbiome studies of insects [29]. *Wolbachia* and/or *Rickettsia* were especially abundant among some weevils, leaf beetles, and ground beetles, whereas these microbes infected only single members of dung beetles in low frequency. The most interesting results were obtained for Scarabaeidae, the microbiota of which was dominated by Pseudomonadales (mainly *Acinetobacter*) and *Carnobacterium* (Bacilli). These bacteria are known from decomposing organic material (like dung or carcasses). This finding is also consistent with the diet of examined dung beetles. Other studies also supported a high prevalence of Pseudomonadaceae in dung beetles [31, 32]. Moreover, some pathogenic bacteria were found in dung beetles such as *Pasteurella* (Gammaproteobacteria), which are known as zoonotic pathogens and most probably originated from domestic animals (cows, sheep, horses) on which dungs these beetles were collected. Also, other bacteria found mainly in dung beetles, but also in a low share in other hosts, were known as facultative pathogens (e.g., *Pantoea*, *Empedobacter, Enterococcus* and others); unfortunately, the only studies which describe their pathogenesis concern vertebrates (e.g., [61, 62]) and data for invertebrates are missing (it is uncertain if only some insects could serves as vectors for these bacteria or could also suffer from their pathogenesis). The aforementioned description of bacteria living in beetles also explains the high dissimilarity between microbiome beta diversity metrics assessed for particular hosts. Despite a great variation of microbiome diversity within species, species from particular families or trophic guilds expressed much greater distinctiveness in their bacterial communities. Various compositions of bacteria in beetle species also causes significant differences if analyzing beetles on higher organization levels. The highest bacteria alpha diversity was found among members of Carabidae and Scarabaeidae; the lowest, among species of Staphylinidae. Simultaneously, all five examined families of beetles possessed substantially dissimilar microbiota (which is expressed by significant differences in beta diversity metrics among these groups). Similar conclusions—that insect bacterial communities are shaped by host taxonomy—were presented by Colman et al. [28] and Yun et al. [29].

An even bigger association between microbiome and trophic affinity was observed with regard to host diets. Principal coordinate analyses and non-metric multi-dimensional scaling plots showed that members of all three examined trophic guilds (carnivores, herbivores, and detritivores) form distinct units with respect to their bacterial communities. This phenomenon suggests that the trophic affinity of the host is more responsible for microbiome similarities than just host taxonomy (phylogenetic relations). Unfortunately, the sampling structure in this study (high correlation between assignment of beetle species to families and trophic guilds) prevents direct and unequivocal conclusions from being made about the role of phylogeny vs trophy. However, the observed pattern that beetles belonging to the particular trophic guild share their microbial diversity, strongly support the statement that common food resources could be mostly responsible for sharing of bacteria. This is not only consistent with the meta-analysis of insects by Colman et al. [28] and the microbiome study of insects by Yun et al. [29], but also with studies on mammals. Ley et al. [63] showed that taxonomy as well as host diet are correlated with bacterial community diversity in wild and domestic mammals (including humans). Moreover, a study by Muegge et al. [64] proved that microbial communities adapt to extremes of diet and that this is probably the case in beetles.

Indeed, endosymbiotic bacteria are one of the most significant findings of the presented work. So far, only *Wolbachia* has been known to infect numerous taxa of beetles (see [22] for a summary). There were also single examples of infection of *Rickettsia* and *Spiroplasma* in beetles [22, 29]. *Cardinium* has not been detected thus far in any beetle species [20, 29]. There have been no reports about the status of *Arsenophonus* in beetles. Here, thanks to use of next-generation sequencing technology, we have extended the list of endosymbiotic bacteria known from beetle into eight taxa with new finding for six bacteria: *Cardinium* (found only in one species of ground beetle), *Arsenophonus* (found only in one species of ground beetle), *Buchnera* (found in nine hosts), *Regiella* (two hosts), *Serratia* (three hosts), and *Sulcia* (two hosts). All these six, newly detected bacteria were only found in very low frequencies in some individuals of particular beetle species. Therefore, it is not possible to make any conclusions about their potential effects of beetles (this is outside the scope of this article), but it is known that *Buchnera* and *Sulcia* are mutualistic, whereas *Regiella* and *Serratia* are commensalistic [65]. *Cardinium* and *Arsenophonus*, as well as *Spiroplasma*, *Rickettsia*, and *Wolbachia*, were considered to be either parasitic or symbiotic [65]. It is worth mentioning that all beetle species, which have been recently reported to be infected by *Wolbachia* with the use of traditional Sanger sequencing and multilocus sequence typing genotyping [47–49, 60, 66, 67], were also found to be infected in this study (they have a large number of *Wolbachia* reads in Illumina sequencing). It is interesting that the collected data reject the hypothesis that endosymbiotic bacteria from particular genera inhabits hosts interchangeably, which would explain the effect of some competition between taxa having similar effects on the hosts [68] (all these bacteria described above are transmitted transovarially [65]) and all these most abundant are known as “male-killers” [18]. Obviously, these different bacteria could live together in the same host while there are no relations among them, which could change their abundance with respect to prevalence of other bacterial taxa. The lack of a visible effect on relative abundance does not say much about other possible relations between these bacteria in multiple infected hosts. Further research is needed to investigate possible effects among various endosymbiotic bacteria, other microbes and their hosts. Another result worthy of highlighting is that this study only weakly supports the assumption that a higher abundance of endosymbionts decreases diversity of the whole microbiota [3]. Apparently, the association between the number of endosymbiotic bacteria and alpha diversity of other bacteria is not straightforward, and other factors could be responsible for the lower richness of bacteria in, e.g., *Wolbachia-* or *Rickettsia*-infected hosts. Also, this finding should be verified in further research. This last finding is supported by multivariate analyses, which indicates that endosymbiotic bacteria abundance is less responsible for microbiome diversity than phylogenetic relations of infected hosts (expressed by genetic distances among examined beetles).

In summary, this study rejects the hypothesis that overall on the individual level, beetles possessed more similar microbiota within species than between taxa. However, even if most beetle hosts (individuals) vary greatly, there are some beetle species, the members of which are infected by unique bacteria communities. Such communities made their host distinguishable from others. Contrary to the high intermixing of microbiota found on the individual level, there are much clearer differences of bacterial communities found in particular groups of beetles considered on higher taxonomic levels (within family). The greatest distinctiveness was observed among beetles grouped according to their trophic requirements. This finding supports the conclusions of other studies. Microbiome is shaped by both phylogenetic relations (in simplicity—taxonomy) as well as trophic affinity, and that the latter better explains differences among groups of infected hosts. Moreover, this study greatly extends knowledge about endosymbiont prevalence in Coleoptera, supporting infection by not only common *Wolbachia*, *Rickettsia*, and *Spiroplasma*, but also other bacteria such as *Cardinium*, *Arsenophonus, Buchnera*, *Sulcia*, *Regiella*, and *Serratia*. The obtained results do not support the interchangeability of endosymbiotic bacteria in beetle hosts, and suggest only weak decrease of microbiome diversity in taxa highly infected by endosymbionts.

## Electronic supplementary material

ESM 1 (PDF 10.3 mb)

ESM 2 (XLSX 542 kb)

## Data Availability

Sequences produced for this study are available via the Sequence Reads Archive (https://www.ncbi.nlm.nih.gov/sra) under SRA study accessions provided in Table 1.
