# Supplementary material for: How Hosts Taxonomy, Trophy, and Endosymbionts Shape Microbiome Diversity in Beetles
Source: Microb Ecol. 2019 Mar 27;78(4):995–1013. doi: 10.1007/s00248-019-01358-y (PMC6842344; doi:10.1007/s00248-019-01358-y)
Supplement: Supplementary file 1 — (PDF 10.3 mb) [file 248_2019_1358_MOESM1_ESM.pdf]

Supplementary figures to:

How hosts taxonomy, trophy and endosymbionts shape microbiome diversity in beetles

MICROBIAL ECOLOGY

doi: 10.1007/s00248-019-01358-y

Michał Kolasa[1*], Radosław Ścibior[2], Miłosz A. Mazur[3], Daniel Kubisz[1], Katarzyna Dudek[4], Łukasz Kajtoch[1]

[1] Institute of Systematics and Evolution of Animals Polish Academy of Sciences, Krakow, Poland

[2] Department of Zoology and Animal Ecology, University of Life Sciences in Lublin, Lublin, Poland

[3] Institute of Biology, University of Opole, Opole, Poland

[4] Molecular and Behavioral Ecology Group of the Jagiellonian University, Krakow, Poland

* corresponding author: michal.r.kolasa@gmail.com, phone: +48 664404716, ORCID: 0000-0003-2143-429X

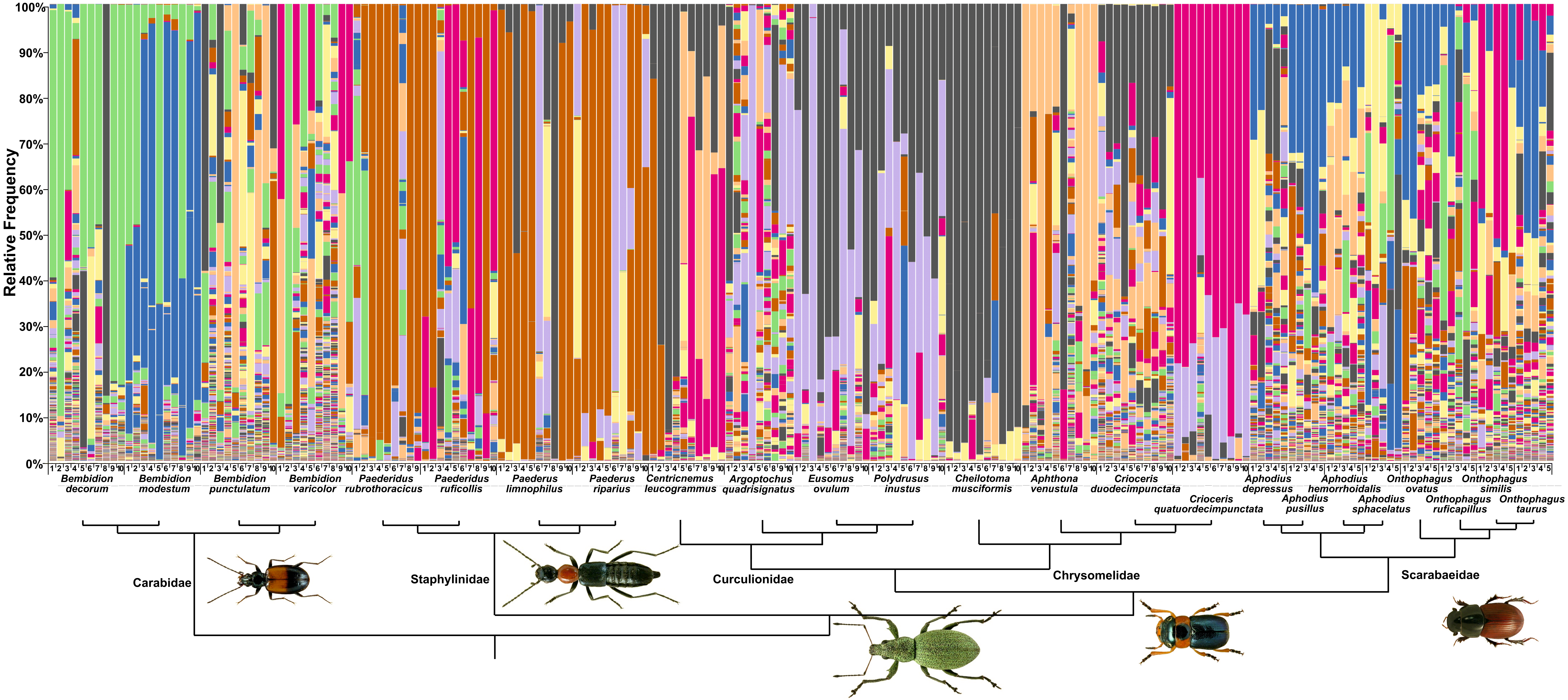

Supplementary figure 1. The plot of the relative share of bacteria in examined specimens of 24 species of beetles showed in relation to a simplified phylogenetic tree of examined beetles. Photographs of exemplary infected beetle hosts are reprinted from ICONOGRAPHIA COLEOPTERORUM POLONIAE under a CC BY license, with permission (© Copyright by Prof. Lech Borowiec, Wrocław 2007–2018, Department of Biodiversity and Evolutionary Taxonomy, University of Wroclaw, Poland)).

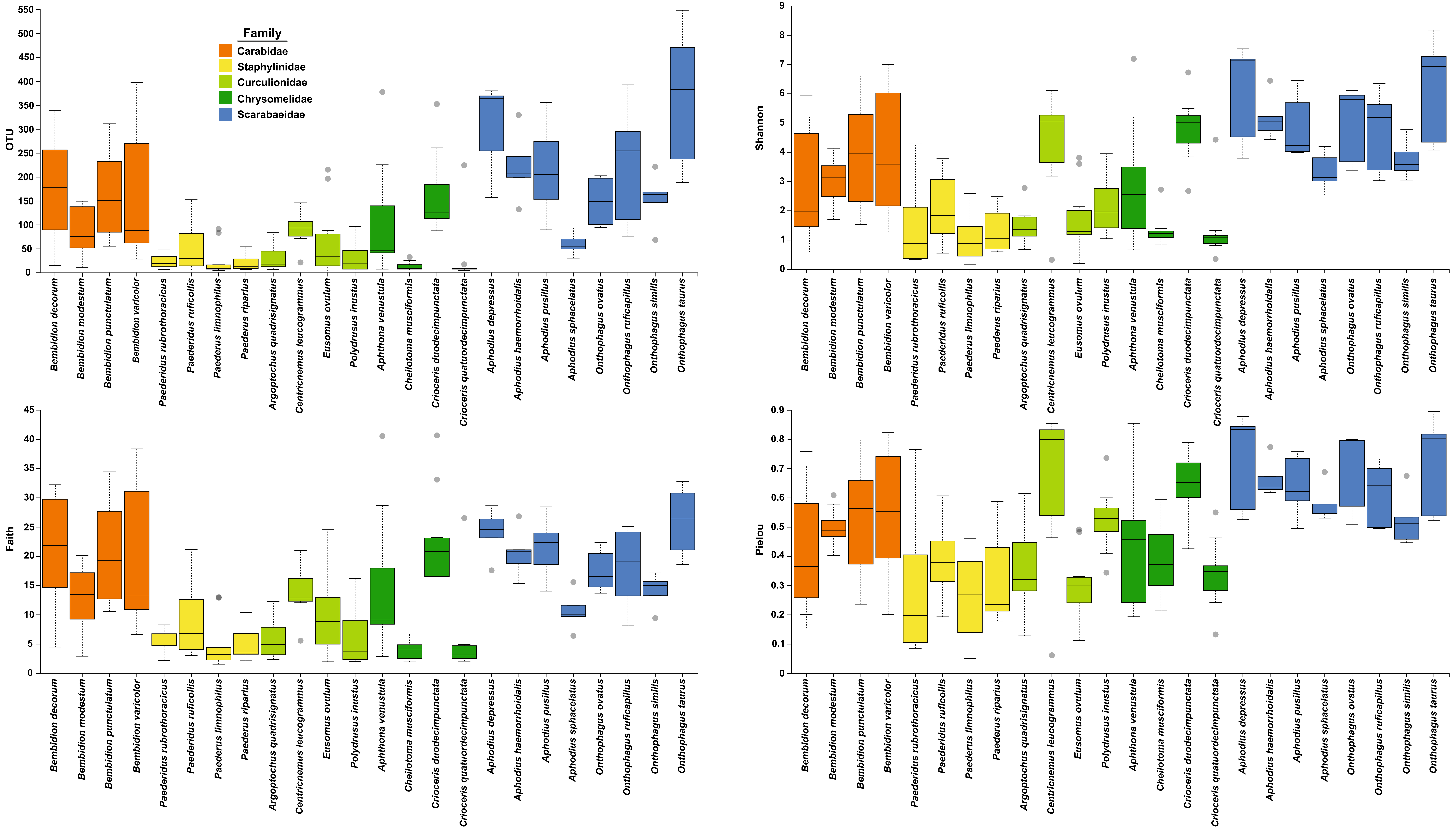

Supplementary figure 2. Box-plots of microbiome alpha diversity metrics (observed Operational Taxonomic Units OTU, Shannon's diversity index - Shannon, Faith's Phylogenetic Diversity - Faith and Pielou's measure of species evenness - Pielou) presented for particular species of beetles.

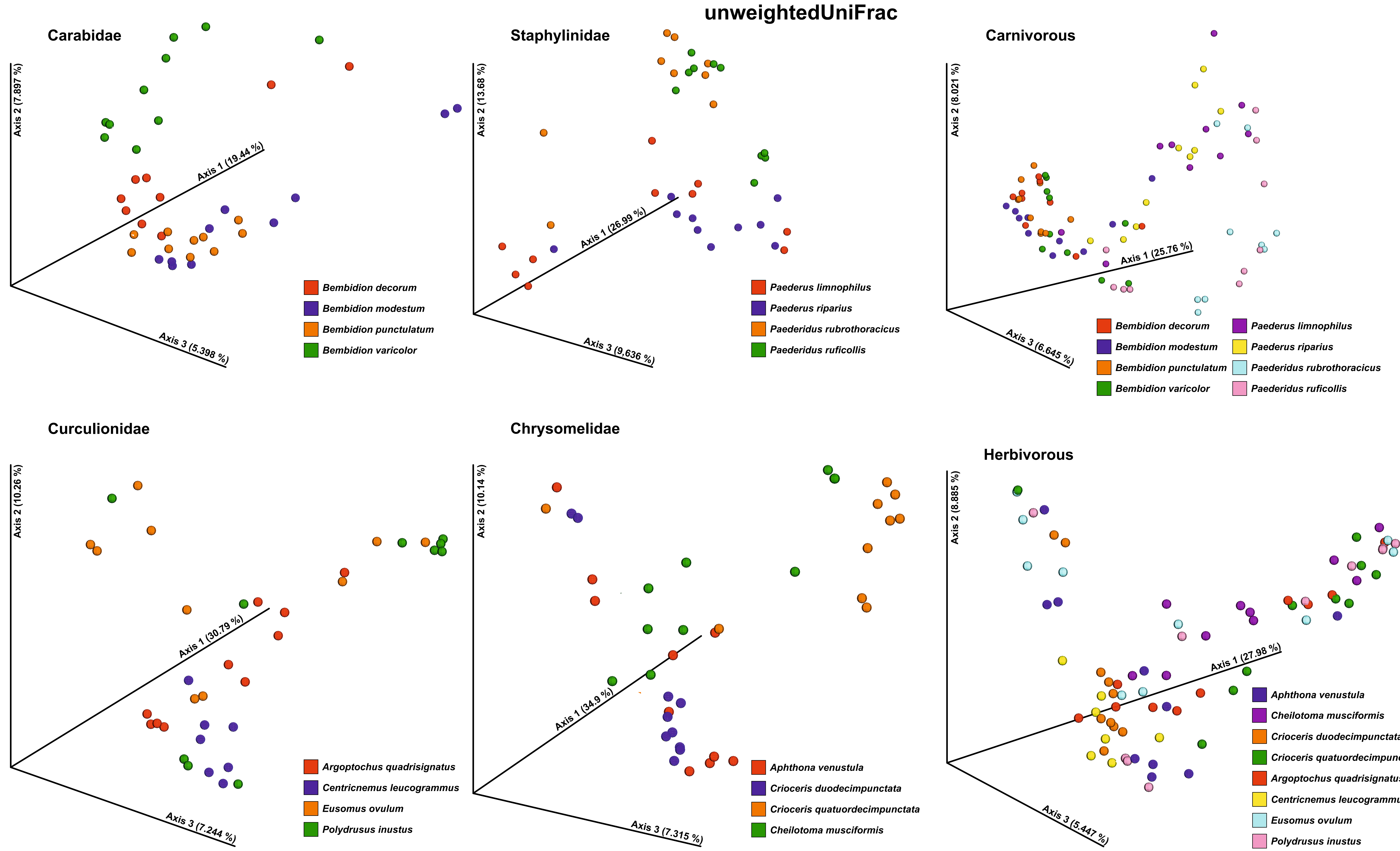

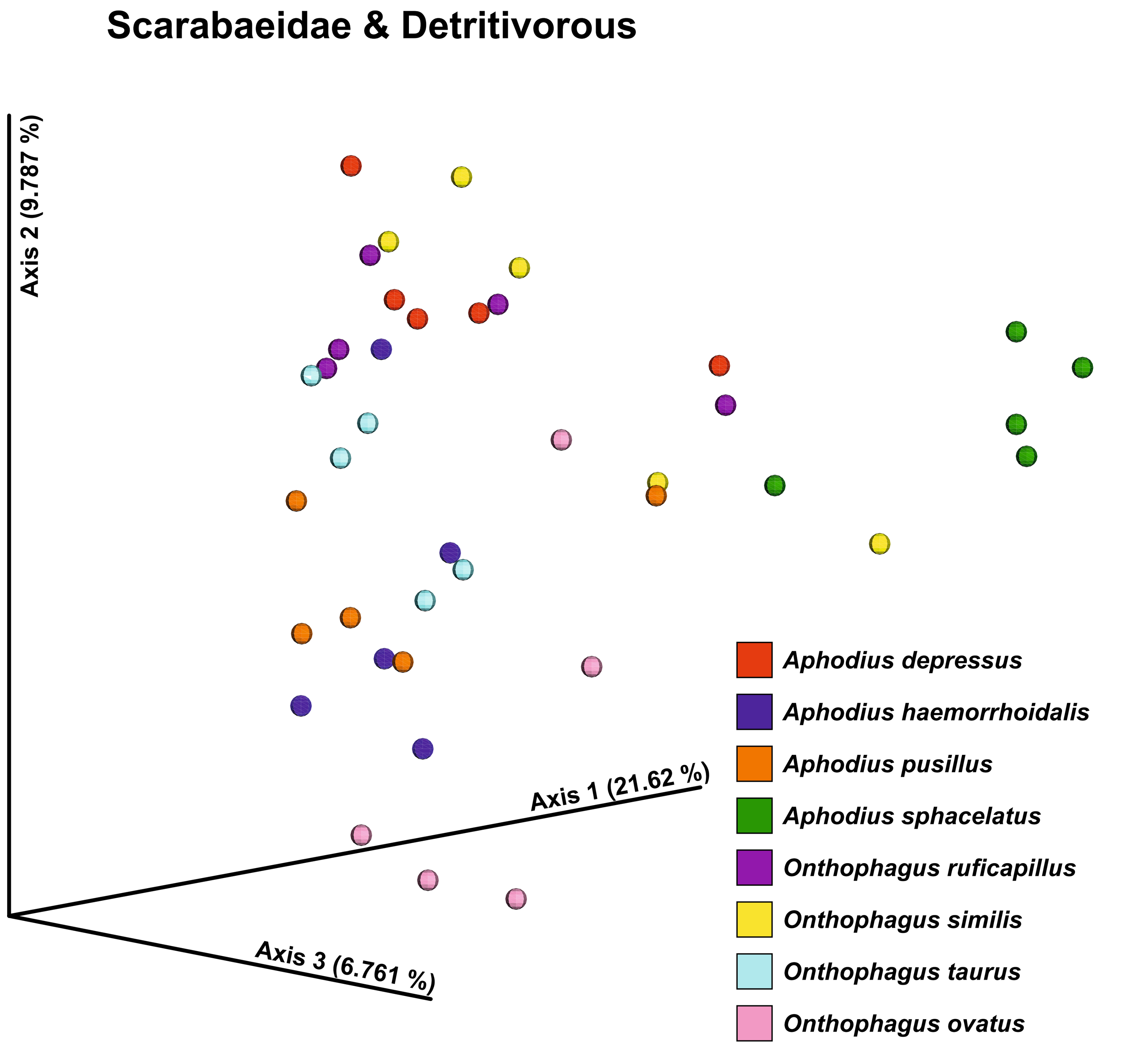

Supplementary figure 3. Principal coordinate analyses of microbiome beta diversity metrics (unweighted UniFrac) presented for particular species of beetles separately for each of examined families and trophic guilds.

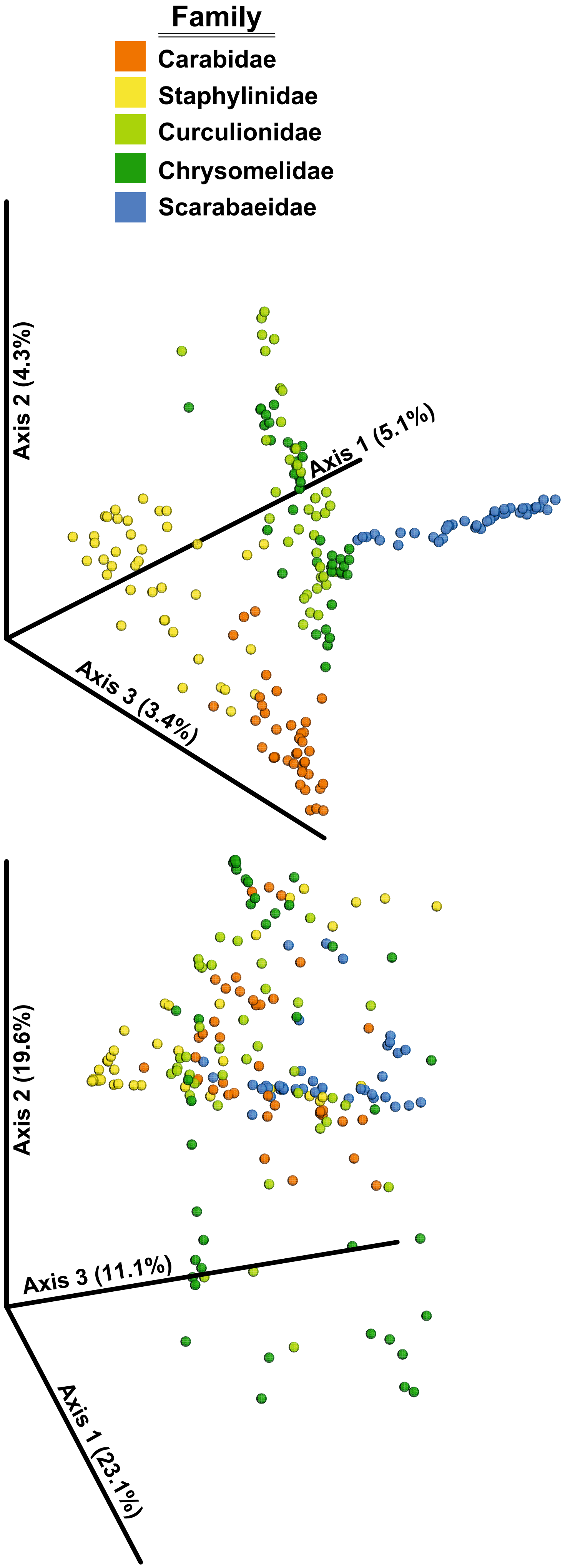

Supplementary figure 4. Principal coordinate analyses of microbiome beta diversity metrics (Jaccard distance – Jaccard and weighted UniFrac - wUniFrac) presented for beetles on the level of families and trophic guilds.

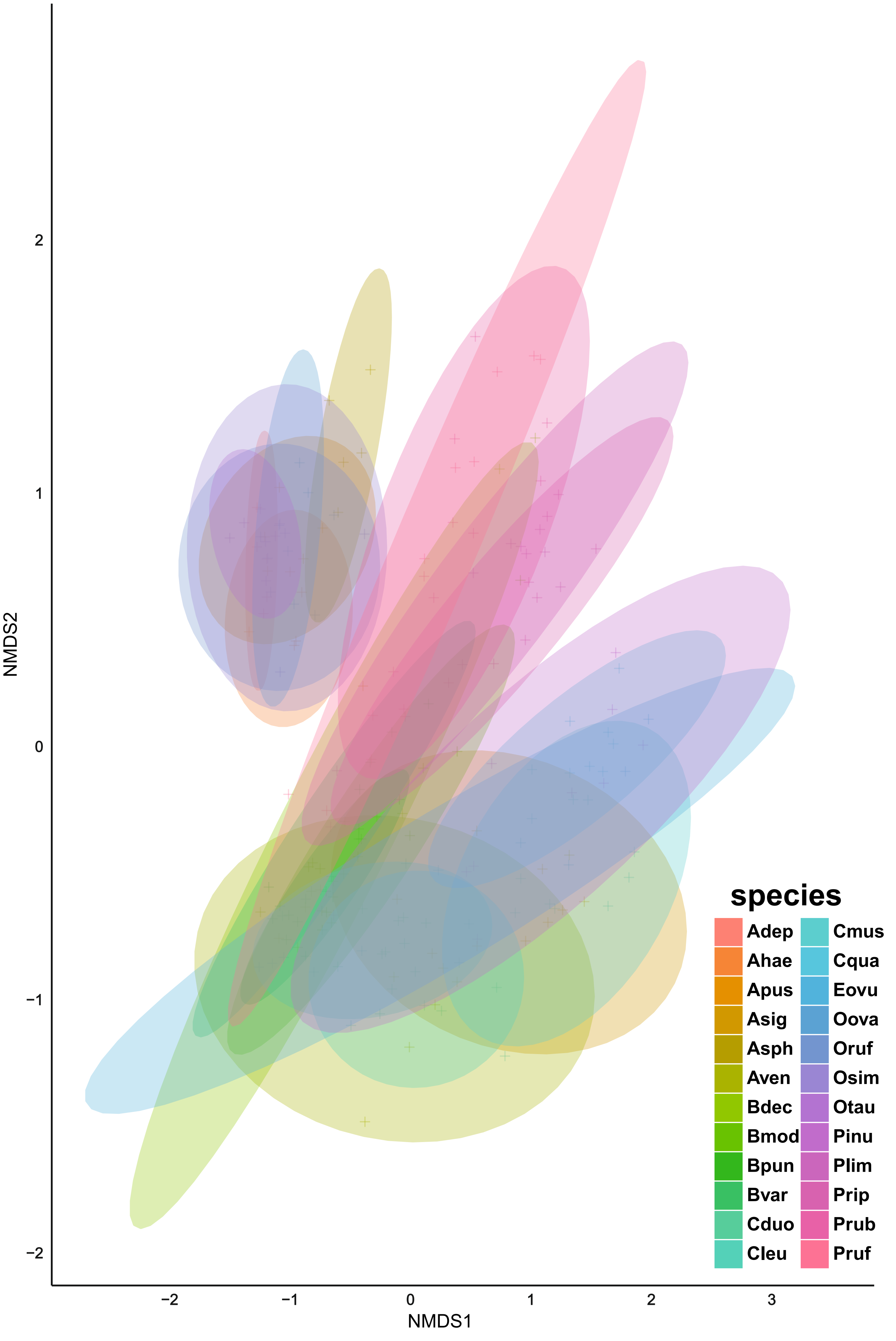

Supplementary Figure 5. Non-metric multidimensional scaling plot of Bray-Curtis dissimilarities between microbiomes found in beetle individuals belonged to particular species. Ellipses – 95% confidence intervals.
